# Interactions Between Policy Effects, Population Characteristics and the Tax-Benefit System: An Illustration Using Child Poverty and Child Related Policies in Romania and the Czech Republic

**DOI:** 10.1007/s11205-015-1083-6

**Published:** 2016-05-05

**Authors:** Silvia Avram, Eva Militaru

**Affiliations:** 1 ISER, University of Essex, Wivenhoe Park, Colchester, CO43SQ UK; 2 National Research Institute for Labour and Social Protection, 6-8 Povernei Street, Sector 1, 010643 Bucharest, Romania

**Keywords:** Child poverty, Child benefits, Microsimulation, Policy interactions, Population interactions

## Abstract

We investigate the impact of the Romanian and Czech family policy systems on the poverty risk of families with children. We focus on separating out the effects of policy design itself and size of benefits from the interaction between policies and population characteristics. We find that interactions between population characteristics, the wider tax benefit system and child related policies are pervasive and large. Both population characteristics and the wider tax-benefit environment can dramatically alter the antipoverty effect of a given set of policies.

## Introduction

The past 20 years have witnessed prominent policy initiatives to tackle child poverty both at the European and national levels (for example, the Lisbon strategy or the Labour government pledge to halve child poverty in the UK by 2020). However, despite these efforts, child poverty rates have remained stubbornly high. Even more worryingly, they have increased in some countries especially in comparison with overall poverty rates (Oxley et al. 2000; Van Mechelen and Bradshaw 2013). For example, between 2005 and 2012, poverty among children in the 27 Member States has broadly remained stable around 28 % whereas poverty among the population as a whole fell from 26 to 25 % (EUROSTAT).

A large body of scholarly work has linked poverty, and low income in general, to deleterious consequences on child developmental trajectories and educational attainment (Black et al. 2000; Engle and Black 2008; Najman et al. 2009; Petterson and Burke Albers 2001), health status (Aber et al. 1997; Case et al. 2002), as well as adulthood outcomes (Duncan et al. 1998, 2010).

Given the consequences of material deprivation both on current well-being and future capability and the fact that children generally have little control over what economic resources are available to them, there is overwhelming agreement that child poverty is an area necessitating public intervention. To mitigate child poverty, governments can resort, among other tools, to various forms of income support and child contingent transfers.

Previous scholarly work has found considerable evidence that child contingent transfers do have a substantial effect on child poverty outcomes, with typically large cross-national variation in policy effects (Matsaganis et al. 2007; Barrientos and DeJong 2006; Bradshaw 2006; Immervoll et al. 2000; Whiteford and Adema 2007). These studies usually use either pre-transfer post-transfer comparisons or a microsimulation-based approach and attribute any differences in observed poverty or inequality indicators to the policy package they investigate. One aspect left unaddressed in these studies is the extent to which policy effects are shaped by ‘outside’ factors, i.e. population characteristics and/or the wider tax-benefit system in which they operate. Although these studies generally acknowledge the existence of interactions of various sorts and their potential in shaping the impact of family transfers, they fail to explicitly investigate these issues. As a result, there is little evidence on the sensitivity of estimated policy effects to variation in the population profile and the design of other social and fiscal instruments that are present. For example, can these factors alter the ranking of policy instruments with similar objectives? These issues are all the more important as the European Union (EU) has launched various benchmarking exercises that essentially rely on comparisons between countries with potentially very different demographic, labour market and tax-benefit institutions.

This paper seeks to bridge this gap and contribute to the understanding of the role of interactions between child contingent policies, population characteristics and the wider tax benefit system in shaping the impact of the former on child poverty. By interactions, we mean that the magnitude of the policy effect is itself contingent on other factors, in particular population characteristics and/or the architecture of the wider tax-benefit system. To this end, we take Romania and the Czech Republic as case studies and examine the reduction in child poverty effected by three family transfers and one tax concession (see Table 1). Romania is a country with high levels of child poverty where the support package available to families with children has been found to be not very effective (TARKI 2010). In contrast, the Czech Republic registers low overall and child poverty rates which have been found to be at least partly the result of generous income support (TARKI 2010). Using microsimulation techniques, we examine to what extent these results are driven by the characteristics of the child-related policy instruments themselves as opposed to being the product of the ‘fit’ between these instruments, other income support measures available to families with children and population features. More specifically, we compute the direct, first-order effect of both the Romanian and the Czech child policy package on relative poverty, while varying the underlying population characteristics and the wider tax-benefit system. Following Salanauskaite and Verbist (2013), we also distinguish between instrument generosity and instrument design in measuring the impact of a given child policy package. The rest of the paper proceeds as follows. Section 2 reviews the existing literature on the links between child related transfers and child poverty. Section 3 describes the Romanian and Czech policies we consider in this exercise. Section 4 describes the data and methods. The various counterfactual scenarios we simulate are explained in Sect. 5. Section 6 discusses our main results. Section 7 concludes.

**Table 1:** Policy instruments included in the child package

Policy	Eligibility	Amounts	% Of children in families receiving^a^	Average amount as % of HH disposable income^b^
*Romania*				
Allowance for new born children and the outfit for new born children	Universal entitlement for all new-borns	Lump sum of approx. 354 RON	6	2
Universal child benefit	Age <18 or in high school	Per month/200 RON for children under 2; 25 RON for children 2 and older	100	7
Means-tested family benefits	Means-tested; monthly income <176 RON per person; children are persons <16 or <18 and with family income <50 RON/month	Between 36 and 52 RON/month, depending on the number of children for 2 parent families and between 52 and 79 RON per month for single parent families	40	9
Tax allowance for dependent children	All employed parents with employment income below 3000 RON/month; the tax allowance is only deductible against employment income; children are considered dependent if aged <16 or having an income below 250 RON/month	Maximum 100 RON per child, max 400 RON/month. The tax allowance is reduced on a sliding scale between 1000 and 3000 RON per month; it reduces to zero once gross employment income reaches 3000 RON/month	69	2
*Czech Republic*				
Child allowance	Means-tested; family income is <4 times the family minimum living standard level; children are individuals younger than 18 or younger than 26 and in education	Between 16–36 % of the child’s minimum living standard (which depends on age), depending on family income	74	3
Social allowance	Means-tested; income is <2.2 times the family minimum living standard; children are individuals younger than 18 or younger than 26 and in education	Child’s minimum living standard from which a share may be deducted based on the size of family income relative the family’s minimum living standard level	27	6
Birth grant	Universal entitlement for all new-borns	Lump sum between 17,760 and 79,680 CZK, depending on number of children in the family	9	5
Refundable child tax credit	Universal entitlement for all parents with dependent children; the tax credit is only refundable if employment income is larger or equal to 6 minimum wages/year	6000 KCZK/month per child, up to a maximum of 5 children	90	3

Children are considered to be individuals aged 17 or less; all policies refer to 2007

*Source*: Authors’ compilation based on EUROMOD G1.4

^a^Percentages calculated based on simulated entitlements in EUROMOD, not on actual reported receipt in SILC

^b^Figures calculated based on households receiving only; in the case of tax concessions, figures are based on approximations not exact amounts

## Child Poverty and Public Transfers: A Review of the Literature

There is a long literature trying to evaluate the role of social and fiscal policies on the welfare of families with children (Gornick and Jäntti 2010, 2011; Jäntti and Bradbury 2003; Barrientos and DeJong 2006; Figari et al. 2011; Whiteford and Adema 2007; Oxley et al. 2000; Sutherland and Piachaud 2011; Bradbury and Jäntti 2001). These studies usually compare poverty and inequality indicators based on market incomes alone with the same indicators derived based on disposable incomes and find that taxes and transfers play an important role in reducing poverty among families with children, although there is considerable cross-national variation in the extent of this reduction. For example, examining child poverty rates among high income countries, Gornick and Jäntti (2010, 2011) conclude that cross-national variation is explained not so much by demographic factors as by labour market institutions alongside the existing system of transfers.

Similar exercises have been carried out using child related policies (Van Mechelen and Bradshaw 2013; Matsaganis et al. 2007; Förster and Tóth 2001; Immervoll et al. 2000; Bradshaw 2006). Generally, these studies find that transfers targeted at families with children significantly reduce both the prevalence and the depth of child poverty, albeit the size of the reduction varies substantially across countries.

Studies directly looking at infant outcomes such as birth weight and neonatal mortality rates also find positive effects of income support availability to disadvantaged women and parents (Hoynes et al. 2011; Almond et al. 2011). Finally, the availability of income support has been found to positively affect not only outcomes measured during childhood but also long run outcomes such as health and economic self-sufficiency in adulthood (Hoynes et al. 2012).

Although there is general consensus that directing resources to low income families with children is a good way to invest in the next generation, there is less agreement on what aspects make a policy more effective. Some authors stress the size of the transfer package (Notten and Gassman 2008; TARKI 2010). According to this view, it is mainly the generosity of the transfer system towards families with children that is likely to impact on child poverty rates. However, public child contingent support is rarely equally generous towards all families with children. Explicitly or implicitly, policy instruments are likely to favour families with some characteristics and not others (ex: number and age of children, number of adults/earners in the household, family income, tax-paying status etc.). Clearly, the effect of a given set of policies on child poverty depends to a large extent on the demographic and labour market characteristics of poor families.

A different strand in the field has argued that in addition to size, policy design plays an important role in determining policy effectiveness (Salanauskaite and Verbist 2013; Levy et al. 2009; Immervoll et al. 2000). Generally, these studies have relied on cross-national comparisons, and/or microsimulation methods to measure the impact of policies, as well as to estimate the effect of alternative policy designs. Although providing important insights into the importance of policy design, these studies usually stop at concluding that one set of policies would likely have been more effective than another in a particular context. There is little potential to generalize *what features* of the design are likely to make a policy more effective than another. More importantly, they fail to consider the sensitivity of the results to the demographic and wider institutional context they have been derived from.

Finally, a large body of the literature has focused on the role of targeting transfers in general and family benefits in particular in addressing poverty (Atkinson 1995; Jarvis and Micklewright 1995; de Neubourg et al. 2007; Förster and Tóth 2001). While some authors (Nelson 2004; Korpi and Palme 1998) have found evidence of a negative correlation between targeting and the overall budget available for public transfers (the famous redistribution paradox), it is not clear that this relationship holds when child related policies are concerned. On the contrary, countries that combined universal benefits with targeted support for low income families with children appeared to achieve superior poverty reduction (Van Mechelen and Bradshaw 2013).

To sum up, extensive research in the area of child poverty consistently finds that public transfers can play an important role in shaping poverty outcomes for families with children. Nonetheless, we still understand relatively little about which aspects of transfer policies, beyond size, matter most and how these interact with demographic characteristics and the wider fiscal institutional context in which they operate. This paper begins to address this gap by examining the extent to which policy impacts are shaped by the characteristics of the population they apply to and the tax-benefit system within which they operate. We address two questions. First, we assess the variation in estimated policy effects when the context, i.e. population characteristics and/or the tax benefit system, changes. Can the same set of policies produce very different estimates when the context is altered? Second, we probe whether the ranking of policy instruments is context specific. For example, is it possible that one set of policies is more effective in the context of the Romanian population but a different set of policies is most effective in the context of the Czech population?

## Child Poverty and Child Support in Romania and the Czech Republic

**Fig. 1:**
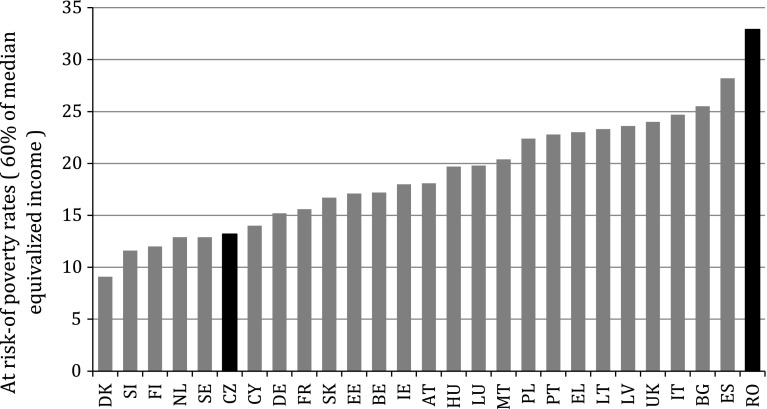
Child poverty rates in the EU, 2007. *Note* Children are defined as aged 17 or less. *Source* EUROSTAT database, http://epp.eurostat.ec.europa.eu/portal/page/portal/statistics/search_database

From a historical perspective, Romania and the Czech Republic share a number of similarities. Both countries have experienced during half a century a foreign-imposed regime based on a command economy combined with suppression of political and civil freedoms. During the nineties, both countries have undergone an extensive political and economic transition that ended with becoming full members of the European Union in 2004 and 2007 respectively. Despite these similarities, the two countries differ in a number of important respects. In particular, the Czech Republic is much richer with a GDP/capita in 2012 of approximately 20,700 PPP compared to Romania’s 13,500 PPP (EUROSTAT). It is also a country with less inequality as measured by the Gini coefficient (25 vs. 33, EUROSTAT). Most importantly, for our purposes, the two countries rank very differently on child poverty indicators (see Fig. 1). While in the Czech Repulic poverty rates for chidlren are relatively low in comparative perspective, Romania is one of the EU Member States with the highest prevalence of child poverty. Finally, the Czech Republic has slightly higher levels of taxation compared to Romania (40 % of GDP is collected in taxes in the Czech Republic versus 35 % in Romania) and spending on cash social transfers (12.5 % of GDP compared to 9.2 % of GDP in Romania) (EUROSTAT).^1^

Since family benefits have been shown to be able to significantly influence poverty and inequality (see Sect. 2), the large discrepancy in child poverty outcomes may partly be explained by differences in child related public transfers. Obviously, children can be made better off through a variety of public measures benefiting their families, ranging from income transfers, to tax advantages and concession, to subsidies or in-kind provision of goods and services.^2^ In this paper however, we limit our attention to transfers and tax concessions directly linked to the presence of children. Using 2007 as our reference year,^3^ we isolate four^4^ policies in each of the two countries (three transfers and a tax concession) which we collectively term the child support package and which will form the focus of our analysis in the remainder of the paper. Table 1 provides an overview of the main policy elements.

In 2007, Romania had three child related benefits. The first is a universal, flat-rate child allowance that covers all children younger than eighteen and pays the same amount irrespective of birth rank. There is however substantial age related variation. Children under two benefit from an increased allowance approximately eight times higher than the one available to older children. In fact, the level of the benefit for young children is unusually high, representing approx. 16 % of the average gross wage in 2007. On average, this benefit constitutes approximately 7 % of household disposable income for the families that receive it. Low income families with children may be entitled to a supplementary allowance. Entitlement is subject to passing an income test which is fairly stringent. The benefit amount depends on the number of children present in the household. However, the benefit increases less than proportionally for higher rank children and is capped after the fourth child. Lone parent families are subject to the same income test but are entitled to higher benefit rates. In total, approximately 40 % of children live in families receiving this type of transfer. For households that receive it, the means-tested family allowance represents approximately 9 % of household disposable income. Finally, in 2007, Romania also had a birth grant which was a lump sum payment to all new-borns equal to approximately 28 % of the average gross monthly wage (or 2 % of household disposable income for the families that receive it). In addition to these transfers, families with children also qualify for tax relief in the form of a tax allowance on employment income. The level of the tax allowance is relatively low and its value is further decreased by the low rate of the personal income tax. Receipt of the allowance is income tested and the amount decreases on a sliding scale depending on the value of taxable earnings. Although the tax allowance is available to all employed parents with low and moderately high employment incomes, only 69 % of children live in families that benefit from this allowance. For these families, the gain due to the presence of the tax allowance represents approximately 2 % of household disposable income.

In the Czech Republic, the main child benefit is income —tested. Receipt is restricted to families with an income less than four times the family minimum living standard (MLS). Nonetheless, the income conditionality is largely designed to exclude wealthy families rather than restrict transfers to the poor. This aspect is confirmed by the fact that 74 % of children receive this benefit (see Table 1). The benefit amount depends on the child’s age (older children are entitled to increased payments) as well as on family income (families with lower incomes are entitled to more generous rates). Overall, for recipient households, the average benefit amount equals approximately 3 % of household disposable income. In addition to the main child benefit, low income families may be entitled to an additional income-tested transfer, called social allowance. As in the case of the main child benefit, entitlement and benefit amounts are calculated using the family and child MLS levels. However, eligibility is restricted to families with incomes below 2.2 times the family MLS and any family income reduces the value of the benefit. Only 27 % of children live in households receiving this benefit. On average, the benefit represents 6 % of disposable income for the households that receive it. Similarly to Romania, the Czech Republic has a lump-sum grant payable to all new-borns. However, unlike Romania, the benefit increases with higher order births. It is also more generous than in Romania amounting to, on average, 5 % of disposable income for the families that receive it. Lastly, families with children are entitled to a refundable child tax credit. It is refundable only to parents with sufficiently high employment income. The tax credit is the same for all children in the family, irrespective of age and birth rank. Almost all children (90 %) benefit from it with recipient households relying on it for approximately 3 % of their disposable income.

To sum up, the two child support packages are relatively similar. Both feature some universalistic elements (the universal child benefit and the child tax allowance in Romania and the child tax credit in the Czech Republic) together with some means-tested components directed at families with few resources (the means-tested family benefit in Romania and the social allowance in the Czech Republic). Means-testing is somewhat more prevalent within the set of Czech policies. Yet, eligibility thresholds are high enough to allow a significant number of families with children to become entitled.

## Data and Methods

To examine the interaction between child policies, the tax-benefit system and population characteristics, we make extensive use of microsimulation techniques to generate a number of counterfactual scenarios (see the next section for a detailed overview). We then compare child poverty measures under these difference scenarios. To carry out our simulations, we use EUROMOD,^5^ the-EU wide tax-benefit microsimulation model (Sutherland and Figari 2013). EUROMOD combines individual and household data from the EU-Survey of Income and Living Conditions (EU-SILC) with detailed information on social and fiscal national legislation to accurately simulate a wide range of transfer entitlements and tax liabilities at the micro-level. We use the Romanian and Czech components of EUROMOD to simulate all counterfactual scenarios. All our results refer to the policy year 2007 and use the 2008 EU-SILC as the underlying micro data. As SILC 2008 contains income information corresponding to the year 2007, there is no time discrepancy between our policy year and our data year. All the simulations assume full compliance with taxes and full take-up of benefits.^6^ As a result, simulation results refer to the *intended* rather than actual policy impacts.

We define children as individuals aged 17 or less, irrespective of their educational or labour market status. Although children may be considered dependent (and thus entitled to child related transfers and tax concessions) up to much older ages in both countries (subject to additional conditions being staisfied), we have opted to circumvent potential incongruities in the way children are defined across countries and across policy instruments by restricting the age range.

Given our interest lies mainly in the anti-poverty potential of child related transfers and tax concessions among families with children, we need to operationalize poverty. We adopt the current established practice and define poverty in a relative way, based on equivalised household disposable income. Disposable income is calculated as market income plus public transfers minus taxes and social insurance contributions. We use the ʽmodified OECD’^7^ equivalence scale to account for differences in household size as well as economies of scale in consumption. We assume income pooling across household members and attribute equivalised disposable income to each individual, including children.^8^ Poverty is operationalized as having an equivalised disposable income lower than 60 % of the median. To check the sensitivity of our results, we use a second, more stringent, threshold set at 40 % of median equivalised disposable income. We use the term severe poverty to denote poverty defined using the lower income threshold. In all cases, we measure the impact of child related policies on a set of three poverty indicators belonging to the Foster-Greer-Thorbecke (FGT) family (Foster et al. 1984). More specifically, we compute the relative reduction in the poverty rate (FGT0), gap (FGT1) and severity (FGT2).

Finally, we conclude this section with two caveats. First, to keep the complexity of our analysis manageable, we abstract from any behavioural changes triggered by replacing one set of policies with another. From a policy perspective however, behavioural responses clearly cannot be ignored. Second, we do not consider the issue of policy administration costs. For example, it has long been acknowledged that administering targeted benefits is much more burdensome compared to administering universal ones, albeit the difference will depend on many factors such as the incentives to comply, the professionalization of the service administering delivery etc.

## Overview of Policy Scenarios

To quantify policy effects, most scholarly work compares poverty indicators using income before and after the transfers that are of interest. For example, Table 2 shows poverty rates and the mean poverty gap for children using market and social security replacement incomes^9^ (before transfers) as well as disposable income that includes all other transfers (after transfers), including child related ones. Based on these figures, one may conclude that non-contributory and means-tested benefits are more effective in reducing child poverty in the Czech Republic compared to Romania, irrespective of which poverty indicator is used. What this type of comparisons cannot tell us is the extent to which the Czech policies would be similalry effective in a different context. More specifically, if the characteristics of the population and/or the wider tax-benefit system in which they operate changed, would the Czech policies still achieve the same impressive level of poverty reduction?

**Table 2:** Child poverty indicators before and after transfers

	Child poverty rates (%)				Child poverty gap (%)			
	60 % Of median eq. DPI		40 % Of median eq. DPI		60 % Of median eq. DPI		40 % Of median eq. DPI	
	Before transfers	After transfers	Before transfers	After transfers	Before transfers	After transfers	Before transfers	After transfers
RO	31.30	31.05	21.03	16.8	51.31	34.28	52.49	24.45
CZ	17.6	9.6	9.3	1.5	43.53	20.31	55.1	13.5

*DPI* disposable income; market incomes include replacement income from the tax-benefit system such as pensions, sickness benefits, unemployment benefits etc

*Source*: Author’s calculations based on EU-SILC 2008 and EUROMOD G1.4

More generally, we are interested in the role and interconnections between three distinct elements, namely population characteristics, the features of the tax-benefit system and the policies contained in each country’s child support package. By population characteristics we mean all individual or household characteristics that can affect tax liabilities or benefit entitlements. They include demographic characteristics (e.g. age, household composition, gender), labour market characteristics (e.g. employment status, hours worked, occupation) and all market incomes. They also include a small number of transfers that are normally considered part of the tax-benefit system but are not simulated by EUROMOD,^10^ most notably pensions. These elements are taken directly from the underlying EUROMOD input datasets. In using the term ‘tax-benefit system’ we refer to sum of tax and benefit policies simulated in EUROMOD. These include social insurance contributions, income tax, contributory unemployment benefits as well as universal and means-tested transfers [see Münich and Pavel (2012); and Stroe et al. (2012) for a complete description of what is simulated in EUROMOD in each country]. Finally, the child support package is the sum of the four child related policies described in Sect. 3, as simulated by EUROMOD. To gain a better understanding of how each element affects the others, we simulate all possible combinations between population characteristics as captured by the data (Romanian and Czech), tax-benefit system (Romanian and Czech) and child policies (Romanian and Czech—standard and budget neutral).

In each country, in addition to the existing systems in 2007, we simulate three types of policy counterfactuals. To proxy for the income distribution that would be observed in the absence of child related transfers (i.e. pre-transfer income), most research simply uses disposable income minus these transfers. This approach assumes that all the other elements of the tax-benefit system would remain unchanged once child related transfers are eliminated. This assumption is however questionable as many elements of the tax-benefit system are income dependent. For example, if child related transfers are included in the means-test of general social assistance, removing child related transfers would make some families eligible for higher social assistance payments. These adjustments would be automatic. Thus, using disposable income minus child related transfers to approximate the pre-transfer income distribution will usually overestimate the impact of the child related policy package. To address this problem, in our first simulated policy counterfactual, we remove the existing child support package and re-calculate disposable incomes, allowing other elements of the tax-benefit system to react to the changed circumstances of previously eligible families. This scenario provides us with a benchmark against which all policy effects are measured. By comparing it with the original systems, we obtain the net additional effect of the existing child support package on child poverty, conditional on the original population characteristics and wider tax-benefit system.

In the second set of counterfactual scenarios, we introduce the other country’s child related policies, adjusting the monetary parameters in two ways. In the standard policy swap, we transform all monetary policy parameters (income limits, benefit amounts etc.) based on the value of median equivalised disposable income.^11^ This allows us to mirror the generosity of transfers and tax concessions relative to the poverty threshold. Subsequently, we perform a budget-neutral swap where monetary parameters are calibrated so as to keep total aggregate costs constant. Note that budget neutrality is imposed at the tax-benefit system level rather than the child policy package level so as to take into account any potential interactions with the other elements in the system.

Finally, we run the original policy system and the simulated counterfactuals using the other country’s dataset as input. This allows us to understand the role of population characteristics in determining the final policy impact we are interested in. To perform this last set of simulations, incomes in the input datasets are adjusted based on the exchange rate. We also construct a small number of variables needed for the simulations, replicating as much as possible their construction in the other country’s dataset. Table 3 presents an overview of all the simulated policy scenarios. Thus, we obtain 16 income distributions that allow as to investigate interactions as follows.

**Table 3:** Overview of simulated scenarios

Scenario	Data	T-B system	Child policies
1	RO	RO	None
2	RO	RO	RO
3	RO	RO	CZ (standard)
4	RO	RO	CZ (budget neutral)
5	RO	CZ	None
6	RO	CZ	CZ
7	RO	CZ	RO (standard)
8	RO	CZ	RO (budget neutral)
9	CZ	RO	None
10	CZ	RO	RO
11	CZ	RO	CZ (standard)
12	CZ	RO	CZ (budget neutral)
13	CZ	CZ	None
14	CZ	CZ	CZ
15	CZ	CZ	RO (standard)
16	CZ	CZ	RO (budget neutral)

*Source*: Authors’ compilation

First, we start by examining the impact of the child related package on child poverty in the ‘usual’ way. We compare the percent reduction in child poverty indicators achieved by Romanian policies in Romania [Scenario2 (S2) vs. Scenario 1 (S1)] and by Czech policies in the Czech Republic (S14 vs. S13).

Second, to examine the extent to which the impact of child related policies depends on population characteristics, we vary the underlying population while keeping the tax-benefit system fixed. We first examine how the effect of Romanian child policies changes when the characteristics of the population change. We thus compare the effect of the Romanian child benefits (and tax concession) in the Romanian tax benefit system using first Romanian data (S2 vs. S1) and then Czech data (S10 vs. S9). We repeat the same exercise for the Czech child related policies (S3 vs S1 and S11 vs. S9). Finally, we examine the effect of Romanian and Czech policies respectively in the Czech tax benefit system while using Romanian (S7 vs. S5 and S6 vs. S5) and Czech data (S15 vs S13 and S14 vs S13).

Third, we examine the interaction between child support policies and other elements of the tax-benefit system, given population characteristics. To this end, we keep the population characteristics (i.e. data) fixed while we vary the tax-benefit system. We first look at the extent to which the effect of the Romanian child policies on the Romanian population is different when the policies are applied in conjunction with the Romanian tax-benefit system (S2 vs. S1), and with the Czech tax-benefit system respectively (S7 vs. S5). Similarly, we calculate the effect of the Romanian policies on the Czech data using first the Romanian tax-benefit system (S10 vs S9) and then the Czech system (S15 vs S13). Finally, we repeat the same set of calculations for the Czech policies, first examining their effect on the Romanian population (S3 vs S1 and S6 vs S5) and then on the Czech population (S11 vs. S9 and S14 vs S13).

Fourth, we separate child poverty impacts stemming from benefit generosity from those coming from policy design by comparing the effect of introducing the other country’s child support package with monetary parameters adjusted relative to the poverty threshold and relative to the budget size respectively.

Policy effects are calculated as the difference between poverty indicators relative to the scenario when no child policies are present (keeping all the other elements constant). More formally, policy effects are calculated as.

$$PE\left( {\alpha , Pop_{i} ,TB_{i} ,CP_{i} } \right) = \frac{{FGT_{\alpha } \left( {Pop_{i} ,TB_{i} ,CP_{0} } \right) - FGT_{\alpha } \left( {Pop_{i} , TB_{i} ,CP_{i} } \right)}}{{FGT_{\alpha } \left( {Pop_{i} , TB_{i} ,CP_{0} } \right)}}$$

where α = 0, 1, 2 is the FGT parameter; Pop = population characteristics; TB = tax-benefit system; CP = child related policy package, with CP_0_ indicating that no child related policies are present; i = CZ, RO.

## Results

### Interactions Between Policy Effects and Population Characteristics

Table 4 shows the effect of the child policy packages in the Czech Republic and Romania under all data-tax-benefit system combinations. The “standard” approach in the literature covering cross-national comparisons of policy effects is to compare the effect of the policies in the environment from which they originated. In this case, one would compare the effects of the Romanian policies in Romania (column A) with the effects of the Czech policies in the Czech Republic (column H). In this setting, one can conclude that the Czech child related policies are indeed much more effective at poverty reduction among families with children, especially if the higher poverty threshold is chosen. For example, poverty rates (using the higher poverty threshold) are reduced by approximately 38 % by the Czech child related policies, whereas their Romanian counterparts achieve only a 14 % reduction. However, this approach assumes that the policy effect is independent of population characteristics and other policies being present.

**Table 4:** Anti-poverty effects of child related policies across tax-benefit contexts and populations characteristics

Indicators	Effect of RO policies				Effect of CZ policies			
	A S1–S2	B S9–S10	C S5–S7	D S13–S15	E S1–S3	F S9–S11	G S5–S6	H S13–S14
	RO TB sys		CZ TB sys		RO TB sys		CZ TB sys	
	RO pop	CZ pop	RO pop	CZ pop	RO pop	CZ pop	RO pop	CZ pop
*Poverty—60* *% of median income*								
FGT0	−13.86	−35.77	−11.04	−32.24	−15.72	−43.36	−7.84	−38.18
FGT1	−26.87	−46.49	−16.92	−33.81	−33.41	−56.78	−18.81	−38.20
FGT2	−35.47	−56.17	−22.11	−36.26	−44.20	−61.70	−26.41	−38.05
*Severe poverty—40* *% of median income*								
FGT0	−27.40	−69.18	−17.49	−38.80	−34.05	−74.64	−22.38	−36.15
FGT1	−41.15	−72.20	−23.14	−41.96	−52.78	−78.19	−29.01	−44.25
FGT2	−49.45	−76.94	−30.93	−41.01	−60.28	−81.75	−37.41	−43.70

All figures represent percentage reduction in the poverty indicators relative to the scenario when no child related policies are present (keeping population and the tax-benefit system constant); all figures refer to households with children. Each column shows which scenarios are being compared to derive policy effects (ex: the effects in column A are derived as the reduction in poverty indicators between scenarios 1 and 2 relative to scenario 1 (S1–S2)/S1)

*Source*: Authors’ calculations based on EUROMOD G1.4

To test the sensitivity of the child policy effects to population characteristics, one can compare for example columns A and B. These show the effects of the Romanian child policy package when applied to the Romanian population and to the Czech population respectively. It is clear from Table 4 that the Romanian policies are much more effective in reducing poverty among families with children when they are applied to the Czech population. For example, the reduction in the child poverty rates achieved in the context of the Czech population is approximately three times as large as that achieved using the Romanian population. Similalry, the reduction in the rate of severe poverty is more than twice as large when Romanian policies are used with Czech data compared to when they are used with Romanian data. Similarily, the effect of the Czech child policies is much lower when these policies are evaluated using Romanian data (columns G and H or columns E and F). Clearly, population characteristics play a very important role in shaping the impact of policies. Both the Romanian and Czech child support policies are much more effective in reducing poverty and severe poverty when applied to the Czech population, irrespective of the wider tax-benefit system. It appears that features of the Romanian population make it harder to achieve poverty reduction for any set of policies aimed at families with children. This aspect would not be captured if one were to compare policy effects in the “standard” way (comparing columns A and H). More specifically, the much larger anti-poverty effect of Czech child related policies is at least in part due to the characteristics of the Czech population.

### Interactions Between Policies and the tax Benefit System Given Population Characteristics

Next, we examine the interactions between the child support packages available in the two countries and the respective tax-benefit systems. We start with the effect of introducing the Romanian child support policies into the Romanian and Czech policy systems respectively, using first Romanian and then Czech data. As shown in Table 4, the anti-poverty effect of the Romanian policies is somewhat stronger when they are introduced in the Romanian tax-benefit system (columns A vs. C and B vs. D). This is true irrespective of using the Romanian or the Czech datasets and concerns almost all poverty indicators. For example, looking at Romanian policies severe child poverty is reduced by 27 % when introducing the Romanian child support package in the Romanian system but only by 17 % when introduced in the Czech system (columns A and C). Similarly, in the case of the Czech children, severe child poverty is reduced by 69 % when policies are combined with the Romanian tax-benefit system but only 39 % in combination with the Czech system (columns B and D). A similar pattern is observed when analysing the poverty gap or the poverty severity. For example, Romanian child related policies reduce the poverty gap by 41–72 % (depending on population characteristics) when introduced in the Romanian system as opposed to 23–42 % when introduced in the Czech system.

The Czech child related transfers and tax concessions are also generating stronger poverty reduction when used within the Romanian tax-benefit system. Table 4 illustrates the reduction in child poverty indicators when introducing the Czech child-related policies in the Romanian and Czech tax-benefit systems respectively. For example, using Romanian data, poverty in households with children is reduced by 16 % when the policies are introduced in the Romanian system but only by 8 % when introduced in the Czech system (columns E and G). The difference in the effectiveness of the Czech policy bundle appears even stronger when simulations are performed using Czech data. Again, although the differences vary from indicator to indicator, generally, policies are more effective when introduced within the Romanian tax-benefit system rather than the Czech one. To illustrate, severe poverty among families with children is reduced by 75 % when pairing policies with the Romanian system (column F). In contrast, introducing the policies within the Czech system reduces severe poverty by around 36 % (column H).

To sum up, the Romanian tax-benefit system appears to magnify the anti-poverty effects of child income support measures, regardless of population characteristics. Both the Romanian and the Czech child related packages have enhanced effects when applied on top of the Romanian tax-benefit rules. One possible explanation may be that, excluding child related instruments, the Romanian tax-benefit system’s ability to reduce child poverty is lower. As a result of the ineffectiveness of the other instruments in the Romanian tax-benefit system, ‘more poverty’ is left to be dealt with by the child related instruments and hence, the latter appear to be more effective.

### Can Context Alter the Ranking of Policy Effects?

The anti-poverty effect of Romanian and Czech child related policies is highly dependent on the context in which they operate. Both sets of policies are more effective when they operate within the Romanian tax-benefit system and on the Czech population. However, from a policy perspective, it is probably more interesting to find out which set of policies is more effective while keeping the population characteristics and the overall features of the tax-benefit system fixed. In the “standard” comparison where each set of child related policies is assessed using the context where it originated from, Czech child related policies appear to be more effective at reducing poverty among families with children compared to Romanian policies (columns A and H). Whereas the precise level of achieved poverty reduction may vary with population characteristics or the features of the tax-benefit system, are Czech policies *always* more effective than the Romanian ones irrespective of context? When assessing the two sets of policies in the context of the Romanian tax-benefit system, the Czech child policies outperform the Romanian ones on almost all poverty indicators. This result holds when inputting both Romanian (columns A vs. E) and Czech data (columns B vs. F) into the simulated counterfactuals. Thus, given the characteristics of the Romanian tax-benefit system and of the Romanian population, Czech policies are able to effect greater poverty reduction among families with children.

However, when looking in the context of the Czech tax-benefit system, the performance of the two sets of policy packages is very similar. This is the case both when policies are applied to the Romanian (columns C vs. G) and Czech populations (columns D vs. H). In fact, the Romanian set of policies is more effective at reducing poverty rates, gap and severity for some groups such as families with very young children (results not shown).

Thus, the context in which a policy operates can affect not only the absolute magnitude of the estimated policy effects but can also, in some instances, reverse rankings. In our case, the interaction between child related policies and the wider tax benefit system can alter which set of policies is deemed more effective. In the context of the Romanian tax-benefit system, Czech policies are more effective. However, in the context of Czech fiscal and social rules, the two sets of policies generate similar anti-poverty effects, with the Romanian package outperforming the Czech one on some indicators.

### Generosity Versus Policy Design

The last issue we investigate is the role of policy design versus the generosity of the child support package. Admittedly, the size of the transfers/tax concessions is a feature of the policy, and thus could be considered as part of policy design. However, since budgetary resources are not unlimited, it is useful to separate out policy effects due to simply increased spending. For this purpose, in addition to our ‘standard’ policy swaps, we simulate corresponding counterfactuals where all monetary parameters have been adjusted so that the total spending equals the cost of the policies we are replacing (for similar studies see Salanauskaite and Verbist 2013; Levy et al. 2009). Two things should be noted. First, since we are simultaneously replacing four policies, there are potentially many possibilities to obtain a budget neutral counterfactual. We solve this problem by adjusting all the parameters by the same ratio. This strategy also has the advantage that it keeps the relative sizes of the four policies we introduce equal to those in the original system. Second, the budget neutrality is enforced at the tax-benefit system level, not at the policy level. In taking this approach, we account for all budgetary effects generated by interactions between the new policies and the rest of the fiscal and social rules. To give an example, the introduction of more generous child benefits will increase the direct costs. However, if these child benefits are taxable/included in the means-test of other benefits, part of the increased costs will be offset by increased revenue/smaller outlays in other policy areas.

Comparing standard and budget-neutral scenarios of the Czech policies in Romania, the latter are clearly more effective in all dimensions (see Table 5). The differences are rather large for all indicators, averaging around 10 percentage points. While the Czech system relies on income-testing quite a lot, the Romanian one is more universalist with the result that it is generally more expensive. Thus, swapping the Romanian child package for the Czech one and adjusting the monetary parameters based on the values of the poverty thresholds actually costs *less*. Therefore, to achieve budget neutrality, the parameters from the ‘standard’ scenario need to be scaled up by 22 %. As a result, the child benefit package is more generous in the budget neutral scenario and thus achieves better poverty reduction. Coming back to results presented in Table 4, the Czech set of policies (in the ‘standard’ version) outperforms the Romanian one *despite* a lower budget.

**Table 5:** Policy generosity versus policy design: anti-poverty effect of ‘standard’ versus budget neutral policy swaps

Indicators	RO policies in the CZ TB sys		CZ policies in the RO TB sys	
	Standard S13–S15	Budget neutral S13–S16	Standard S1–S3	Budget neutral S1–S4
*Poverty—60* *% of median income*				
FGT0	−32.24	−26.58	−15.72	−21.42
FGT1	−33.81	−42.82	−33.41	−39.68
FGT2	−37.28	−54.28	−41.19	−51.21
*Poverty—40* *% of median income*				
FGT0	−38.80	−69.09	−34.05	−41.96
FGT1	−41.96	−78.65	−52.78	−61.07
FGT2	−41.01	−80.54	−58.93	−67.98

Policy effects have been computed relative to the scenario when no child related policies are present (keeping population and the tax-benefit system constant); all figures refer to households with children. Each column shows which scenarios are being compared to derive policy effects (ex: the effects in the first column are derived as the reduction in poverty indicators between scenarios 15 and 13 relative to scenario 13 (S13-S15)/S13

*Source*: Authors’ calculations based on EUROMOD G1.4

Since the Romanian child package is generally more expensive, its parameters need to be scaled down compared to the standard scenario to achieve budget neutrality. Indeed, the adjustment factor is 0.62 indicating that the needed reduction is quite substantial. Based on this downward adjustment, we would expect the budget neutral swap to perform worse compared to the standard one. Indeed, this is the case when we look at poverty rates defined using the higher income threshold. Nevertheless, differences are small despite the large correction factor. Moreover, both the poverty gap and poverty severity are better mitigated in the budget neutral scenario, despite lowering amounts disbursed via child benefits. In addition, all three poverty indicators show that severe poverty drops much more dramatically in the budget neutral scenario compared with the standard swap. This finding may seem counterintuitive. However, remember that budget neutrality is attained at the system, not at the policy level. It is possible that lower outlays in the form of child related transfers and tax deductions are compensated by increases in other elements of the tax-benefit system. Indeed, disposable income in the first three deciles is virtually unchanged between the two counterfactuals whereas the poverty line is higher (as expected) in the standard scenario (results not shown). This finding highlights (again!) the importance of policy interactions in shaping the overall effect. The capacity of the Czech system in reaching the poor combined the untargeted nature of Romanian policies mean that reducing the latter and increasing parts of the former may lead to better anti-poverty results.

## Discussion and Conclusions

This paper has examined the anti-poverty effect of child contingent policies in Romania and the Czech Republic, paying particular attention to their sensitivity to population characteristics and the wider tax-benefit system they are embedded in. We find that both population characteristics and the other fiscal and social policies exert a substantial influence on policy effects. For example, both the Romanian and the Czech child contingent transfers are more effective when applied in the Romanian tax-benefit system. On the other hand, when applied in the Czech tax-benefit system, both sets of policies have similar effects. This suggests that the Czech tax-benefit system is effective on its own (even in the absence of the child related policies) thus limiting any policy effects coming from the policies themselves. Conversely, the Romanian tax-benefit system has a smaller effect leaving more leeway for policies to have an impact. Thus, policy effects cannot accurately be evaluated independent of the wider tax-benefit system in which they are supposed to operate, as interactions with the other elements of the system are pervasive and play a significant role in determining impact.

We find that quite apart from size, policy design matters on its own. As the case of the Czech policies demonstrates, it is possible to achieve enhanced anti-poverty results on a *lower* budget. Moreover, it is not clear that increasing the size of the transfers will *always* lead to better poverty related outcomes. On the contrary, as shown in the case of the Czech Republic, there may be substitution and trade-offs at the bottom of the income distribution that are less likely to occur in the middle or at the top, especially if targeting is used extensively. As a result, in the absence of co-ordination with other instruments, increased spending on some transfers may be compensated by lower benefits/higher taxes in another area.

Another consistent finding emerging from our analyses is the role of population characteristics. Both the Romanian and the Czech policy packages achieve larger poverty reduction when used together with the Czech population. In our setup, we cannot explicitly disentangle which features of the Czech population are responsible for this result, but we can hypothesize that the much lower inequality of market incomes in the Czech Republic plays a role. If this is the case, it suggests that poverty mitigation is likely to be much harder when the incomes of the poor and the rich are far apart *regardless* of what transfer instruments are in place. It may thus be more efficient for public policies to focus on limiting inequality of market incomes in the first place (through such measures as activation policies, minimum wage setting, steep taxation of very high incomes to discourage their occurrence etc.) rather than trying to direct more resources to the poor via transfers.

Overall, our results point to the importance of interactions between the various policy instruments operating within the same system, as well as to complex linkages between population characteristics and policy design. In principle, the effect of a given set of policies in a particular context cannot be inferred from the effect of the same set of policies in a different context. Unfortunately, these complexities make policy benchmarking and policy learning all the more difficult. What seems to be working very well in one context may not work in another. EU-wide policy reviews recognize these issues explicitly or implicitly when they recommend an ‘appropriate policy mix’ (TARKI 2010). However, what an ‘appropriate policy mix’ should contain still eludes us. Future research should focus on disentangling which population and system characteristics ‘fit’ with which types of policies.

## Appendix: Parameters of the Child Related Policies

See Tables 6 and 7.

**Table 6:** Romanian child related policies

	RO policies in RO system (S2 and S10)	RO policies in CZ system-standard (S7 and S15)	RO policies in CZ system- budget neutral (S8 and S16)
Universal child benefit-amounts	200 RON/month if age < 2; 25 RON/month if age ≥ and age < 18	5628 CZK/month if age < 2; 704 CZK/month if age ≥ 2 and age < 18	3489 CZK/month if age < 2; 436.5 CZK/month if age ≥ 2 and age < 18
Universal birth grant-amount	150 RON/year if age = 0; in addition, 204 RON/year if age = 0 and total number of children ≤4	4221 CZK/year if age = 0; in addition, 5721 CZK/year if age = 0 and total number of children ≤4	2617 CZK/year if age = 0; in addition, 3547 CZK/year if age = 0 and total number of children ≤4
Means-tested family benefit -threshold	176 RON/per month per person	4953 CZK per month per person	3071 CZK per month per person
Means-tested family benefit-amounts	Between 36 and 79 RON per month, depending on number of children and type of household	Between 1013 and 2223 CZK per month depending on number of children and type of household	Between 628 and 1378 CZK per month depending on number of children and type of household
Tax allowance for dependent children-eligibility	Employment income below 3000 RON/month; tax allowance starts being reduced when employment income surpasses 1000 RON/month	Employment income below 84,446 CZK/month; tax allowance starts being reduced when employment income surpasses 28,142 CZK/month	Employment income below 52,357 CZK/month; tax allowance starts being reduced when employment income surpasses 17,448 CZK/month
Tax allowance for dependent children-eligibility	100 RON/month	2814 CZK/month	1745 CZK/month

1 RON = approx. 5.60 CZK (according to June 2007 exchange rates); simulations to which the parameters apply in parentheses

*Source*: EUROMOD G1.4; the conversion of the parameters has been made by multiplying the values in the Romanian system by median household disposable income in CZ/median household disposable income in RO

**Table 7:** Czech child related policies

	CZ policies in the CZ system (S6 and S14)	CZ policies in the RO system-standard (S3 and S11)	CZ policies in the RO system- budget neutral (S4 and S12)
Means-tested child allowance-minimum living standard-adults	Between 2600 and 3126 CZK/month depending on the type of household	Between 92 and 111 RON/month depending on the type of household	Between 112 and 135 RON/month depending on the type of household
Means-tested child allowance-minimum living standard-children	Between 1600 and 2250 CZK/month, depending on age	Between 57 and 80 RON/month, depending on age	Between 70 and 98 RON/month, depending on age
Birth grant amount	Between 17,760 and 79,680 CZK/year depending on birth rank order	Between 631 and 2831 RON/year, depending on rank order	Between 770 and 3454 RON/year, depending on rank order
Social allowance-family minimum living standard	Between 2600 and 3126 CZK/month/adult, depending on household type	Between 92 and 111 RON/month/adult, depending on household type	Between 112 and 135 RON/month/adult, depending on household type
Social allowance- child minimum living standard	Between 1600 and 5400 CZK/month, depending on age and disability status	Between 57 and 192 RON/month, depending on age and disability status	Between 70 and 234 RON/month, depending on age and disability status
Child tax credit amount	6000 CZK/month/child	213 RON/month/child	260 RON/month/child

1 RON = approx. 5.60 CZK (according to June 2007 exchange rates); simulations to which the parameters apply in parentheses

*Source*: EUROMOD G1.4; the conversion of the parameters has been made by multiplying the values in the Czech system by median household disposable income in RO/median household disposable income in CZ

## Footnotes

1 Figures refer to 2007 total government revenue and social benefits other than social transfers in kind.

2 A description of the tax-benefit system in Romania and the Czech Republic in 2007 (our policy year) can be found in the respective EUROMOD country reports (Münich and Pavel 2012; Stroe et al. 2012).

3 While newer policy years were available in EUROMOD at the time this study started, we chose 2007 to match it with the year of our dataset.

4 One important policy instrument that is missing from our analysis are maternal/parental leave benefits; we have opted to exclude them in this case because they are only simulated in Romania.

5 We use version G1.4.

6 We opt to assume 100 % compliance and take-up rates as there is very little information on which modelling of tax evasion and/or benefit non-take-up can be based.

7 The modified OECD equaivalence scale assigns a weight of 1 for the first adult, 0.5 for all subsequent adults and 0.3 for children. Children are defined as being aged 13 or less.

8 This is an often made assumption despite it being innacurate. Some studies (Ward-Batts 2005; Lundberg et al. 1997) have shown that public transfers received by the mother are more likely to be spent on children invalidating the complete intrahousehold redistribution assumption. However, we ignore this issue in this study.

9 Replacement incomes include pensions, sickness benefits, unemployment benefits, maternity and parental leave benefits.

10 Pensions and some smaller transfers such as sickness and maternity benefits are not simulated due to data limitations in the EU-SILC.

11 More specifically, we apply the following formula; new monetary parameter = old monetary parameter* (median equivalised disposable income in the “receiver” country/median equivalised disposable income in the “donor” country); for example when introducing Czech policies in Romania, the new policy parameters in Romania will be = CZ policy parameter*(RO median equivalised household disposable income/CZ median equivalised household disposable income).
